# Dysregulation of the Acrosome Formation Network by 8-oxoguanine (8-oxoG) in Infertile Sperm: A Case Report with Advanced Techniques

**DOI:** 10.3390/ijms22115857

**Published:** 2021-05-30

**Authors:** Sung Woo Kim, Bongki Kim, Jongsoo Mok, Eun Seo Kim, Joonghoon Park

**Affiliations:** 1 Animal Genetic Resources Research Center, National Institute of Animal Science (NIAS), Rural Development Administration (RDA), Hamyang 500000, Korea; sungwoo@korea.kr; 2 Department of Animal Resources Science, Kongju National University, Yesan 32588, Korea; bkkim@kongju.ac.kr; 3 Department of International Agricultural Technology, Graduate School of International Agricultural Technology, Seoul National University, Pyeongchang 25354, Korea; mok4890@snu.ac.kr (J.M.); eun2927@snu.ac.kr (E.S.K.); 4 Institute of Green-Bio Science and Technology, Seoul National University, Pyeongchang 25354, Korea

**Keywords:** 8-oxoguanine (8-oxoG), acrosome, sperm, infertility, bull

## Abstract

8-Hydroxyguanine (8-oxoG) is the most common oxidative DNA lesion and unrepaired 8-oxoG is associated with DNA fragmentation in sperm. However, the molecular effects of 8-oxoG on spermatogenesis are not entirely understood. Here, we identified one infertile bull (C14) due to asthenoteratozoospermia. We compared the global concentration of 8-oxoG by reverse-phase liquid chromatography/mass spectrometry (RP-LC/MS), the genomic distribution of 8-oxoG by next-generation sequencing (OG-seq), and the expression of sperm proteins by 2-dimensional polyacrylamide gel electrophoresis followed by peptide mass fingerprinting (2D-PAGE/PMF) in the sperm of C14 with those of a fertile bull (C13). We found that the average levels of 8-oxoG in C13 and C14 sperm were 0.027% and 0.044% of the total dG and it was significantly greater in infertile sperm DNA (*p* = 0.0028). Over 81% of the 8-oxoG loci were distributed around the transcription start site (TSS) and 165 genes harboring 8-oxoG were exclusive to infertile sperm. Functional enrichment and network analysis revealed that the Golgi apparatus was significantly enriched with the products from 8-oxoG genes of infertile sperm (*q* = 2.2 × 10^−7^). Proteomic analysis verified that acrosome-related proteins, including acrosin-binding protein (ACRBP), were downregulated in infertile sperm. These preliminary results suggest that 8-oxoG formation during spermatogenesis dysregulated the acrosome-related gene network, causing structural and functional defects of sperm and leading to infertility.

## 1. Introduction

In beef production in Korea, more than 90% of the beef is bred by artificial insemination (AI); therefore, the ability to predict the fertility of bulls before AI has been a long-term objective of the animal breeding industry. The reproductive soundness of the selected bulls should be managed. The evolutional conservation of spermatogenesis is reported in the mammalian species. The irregularity of spermatogenic gene expression reduces the quality of semen and may lead to the failure of fertilization both in cattle and humans [1]. Environmental and genetic effects have been associated with decreased male fertility, which is known to contribute to a dysregulated spermatogenic gene network, abnormal spermatogenesis, and eventually, impaired sperm fertility [2]. However, the molecular basis for the cause of poor sperm quality has not been studied in detail.

The integrity of sperm DNA has been recognized to be necessary for the development of normal fertile sperm of mammals, including humans and mice. Sperm abnormality is reported to be caused by reactive oxygen species (ROS), abnormal packaging of sperm chromatin, and cell death [3]. Among them, ROS-induced oxidative stress is well known to play a major role in male factor infertility [4]. Therefore, one of the characteristics of the male reproductive system—low oxygen stress—is recognized as an important component of the self-defense mechanism against oxidative damage during spermatogenesis and hormone production. Along with this, a wide range of antioxidant enzymes and ROS scavengers equipped in the male reproductive system work to ensure that spermatogenesis and steroidogenesis are not impacted by oxidative stress [5]. In contrast, ROS are a prerequisite for spermatogenesis under physiological conditions that contribute to a successful fertilization ability. Sperm, however, are especially susceptible to the harmful effects of ROS on both their cell membranes and DNA. In particular, the spermatids of spermiogenesis are very vulnerable to ROS, as they progressively lose the ability to repair the oxidative DNA damage since haploid spermatids form mature sperm [2].

8-Hydroxyguanine (8-oxoG) is the most common and well-studied oxidative DNA lesion of the four DNA bases; 8-oxoG repair is very important in maintaining DNA integrity because 8-oxoG can be paired not only with cytosine (C) but also with adenine (A) during DNA replication, resulting in a transversion mutation from G:C to T:A base-pairing. Base excision–repair (BER) is the major repair mechanism for these lesions [6,7]. In mammalian cells, 8-oxoG DNA glycosylase 1 (OGG1) is a primary DNA glycosylase that hydrolyzes 8-oxoG to form an abasic site. Apurinic/apyrimidinic endodeoxyribonuclease 1 (APEX1) then binds to this abasic site and cleaves the 5’ end to create a phosphate of sugar at the 5’ end and a hydroxyl group at the 3’ end. Subsequently, to fill the gap, DNA polymerase is recruited through DNA synthesis, and DNA ligase I or III seals the nick and completes the BER process [8].

Unrepaired 8-oxoG modifications have been implicated in various pathogenic conditions, and increased levels of 8-oxoG are associated with DNA fragmentation in sperm [9]. Oxidative stress-induced fragmentation of sperm DNA may damage the paternal genetic contribution to embryonic development [4]. With the progressive loss of the DNA repair ability during spermatogenesis, oxidative DNA damage in sperm is expected to be repaired only after fertilization by maternal BER machinery [10]. However, severe sperm DNA damage can surpass the maternal repair capacity and can have a devastating effect on the subsequent development [11,12]. However, the molecular genetic effects of 8-oxoG generated in sperm DNA by oxidative stress on spermatogenesis are not entirely understood.

Here, we identified one infertile Korean native striped bull at 6 years old, due to asthenoteratozoospermia. We found that 8-oxoG was substantially greater in infertile sperm DNA. To understand whether the increased levels of 8-oxoG induce sperm infertility through gene regulatory effects, we performed comprehensive analyses of DNA and proteins derived from infertile sperm. The majority of the 8-oxoG loci were distributed around the transcription start site (TSS), and 165 genes harboring 8-oxoG, including phospholipases, were annotated exclusively into infertile sperm DNA. Gene set enrichment analysis (GSEA) and the protein–protein interaction networking revealed that the 8-oxoG gene products of infertile sperm participate in the Golgi apparatus among intracellular organelles. Proteomic and immunofluorescence analyses verified that acrosome-related proteins, including acrosin-binding protein (ACRBP) and sperm equatorial segment protein 1 (SPESP1), were downregulated in infertile sperm.

These preliminary results suggest that 8-oxoG formation during spermatogenesis would dysregulate the activity of acrosome-related genes, resulting in structural and functional defects of sperm and infertility. These findings may provide new insights to understand bovine male infertility.

## 2. Results

### 2.1. Characterization of Sperm and Male Reproductive Tract

The relative weight of the male reproductive tract of the infertile C14 bull was substantially decreased compared to the fertile C13 bull. The relative weights of the testes and epididymis of C13 recovered after castration were observed to be 0.442 g/bodyweight (kg) and 0.057 g/kg, respectively, and the relative weights of the testes and epididymis of C14 were 0.200 g/kg and 0.033 g/kg, respectively. Therefore, the testes and epididymis of the infertile C14 were reduced by 45% and 58% relative to C13, respectively.

**Figure 1:**
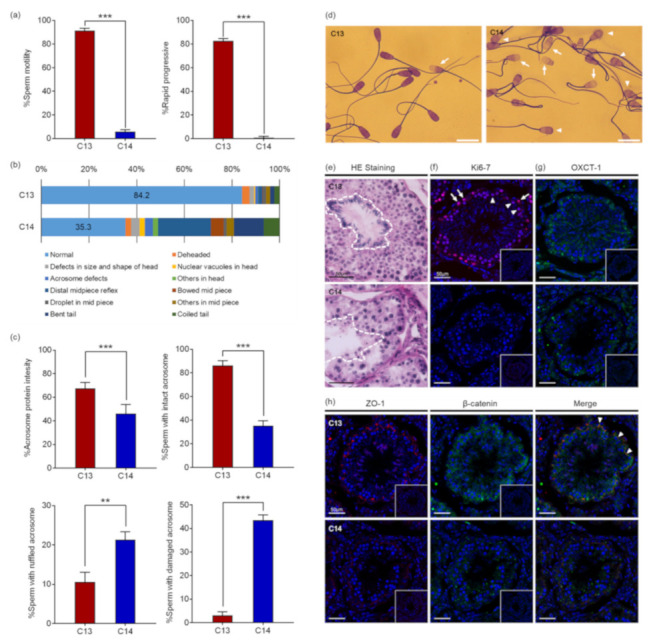
Poor sperm index of infertile C14 sperm. (**a**) Sperm motility and progressiveness by computer-assisted sperm analysis (CASA). Red bars indicate motility and progressiveness of C13 sperm and blue bars for C14 sperm. *** *p* < 0.001 vs. C13. (**b**) Morphological evaluation of sperm. Relative stacked bar chart shows the relative percentage of morphological parameters of sperm in head, midpiece, and tail. Each parameter is depicted in different color. Numbers in the light blue bar indicate the percentage of normal sperm in each group. (**c**) Morphological evaluation of sperm head. Red bars indicate structural parameters of C13 sperm head and blue bars for C14, those including acrosome stain and acrosomal intactness. ** *p* < 0.01, *** *p* < 0.001 vs. C13. (**d**) Representative image of sperm. Arrowheads indicate sperm with ruffled acrosomes and arrows for sperm with damaged acrosomes. Scale bar represents 20 µm. (**e**) Histological comparisons of testes by hematoxylin and eosin (H&E). The area containing elongating spermatid is circled with white dashed lines. (**f**,**g**) Confocal immunofluorescence (IF) microscopy of testis sections from C13 and C14. Anti-Ki67 (red) and anti-OXCT1 (green) antibodies were used. Arrows and arrowheads indicate spermatogonia and primary spermatocytes, respectively. (**h**) Testis was double labeled for zonula occludens 1 (red) and β-catenin (green). Arrowheads indicate co-localization of ZO-1 and β-catenin. Inserts show negative controls. Scale bar represents 50 µm.

Computer-assisted sperm analysis (CASA) revealed that the motility and the progressiveness of C14 sperm were significantly reduced compared to that of C13 (Figure 1a). The analysis indicated that the percentage of motile sperm with standard deviation (SD) of C14 was 5.80% ± 1.67%, which was significantly reduced from 91.28% ± 2.2% of C13 sperm (*p* < 0.0001). In addition, the percentage of progressive motile sperm of C14 was 0.93% ± 0.90%, which was also significantly lower than 82.58% ± 2.06% of C13 sperm (*p* < 0.0001). Therefore, it was determined that the percentage of viable C14 sperm was more than 5.8% and they had severe damage in motility. As a result of sperm morphology analysis, morphologically normal sperm were observed in 84.2% of C13 sperm, whereas only 35.2% of C14 sperm were found to be normal. C14 sperm had higher deformity rates in the head, midpiece, and tail compared to C13 sperm (Figure 1b). About 13.6% of C14 sperm had head abnormalities in the head, including being deheaded, defects in size and shape, nuclear vacuoles, and acrosome defects. In addition, 32.0% of C14 sperm had defects in the midpiece and 19.1% of the tail. Structural abnormalities in the head of the C14 sperm were closely observed through Coomassie Brilliant Blue (CBB) staining (Figure 1c). The percentage of sperm with acrosome stain was significantly lower in C14 (46.2% ± 7.6%) than in C13 (67.5% ± 4.8%, *p* < 0.001). The percentage of sperm with the intact acrosome was also significantly lower in C14 (35.2% ± 4.3%) than in C13 (86.3% ± 4.0%, *p* < 0.001). In contrast, sperm with ruffled or damaged acrosomes were significantly increased in C14 (*p* < 0.01) (Figure 1d). These results suggest that C14 had complete asthenoteratozoospermia.

To investigate the cause of asthenoteratozoospermia, a histological examination of the testes was performed with hematoxylin and eosin (H&E) staining. Contrary to the C13 testes in which seminiferous tubules were filled with organized and defined germ cell layers, seminiferous tubules of C14 were observed with a severe disruption of spermatogenesis (Figure 1e). The obvious spermatogenesis failure was shown by the abnormal organization, amount, and morphology of elongating spermatids. Immunofluorescence (IF) analysis with anti-KI67 antibody was performed to determine if there was a difference in germ cell proliferation (Figure 1f). Proliferating spermatogonia and primary spermatocytes were observed in seminiferous tubules of C13. In contrast, no proliferating cells were observed in seminiferous tubules of C14. To demonstrate whether the spermatogenesis failure detected in C14 was caused by a defect in the nursing function of Sertoli cell, expression of OXCT1, which is restricted to the Sertoli cells and important for energy generation, was investigated (Figure 1g). OXCT1 was highly expressed in the Sertoli cells of C13 compared to C14. The disorganization of spermatogenic cells and defects in Sertoli cells suggested a possible disruption of the integrity of the blood–testis barrier (BTB). To access the integrity of the tight junction and adherens junction of the seminiferous tubules, the expression levels of zonula occludens 1 (ZO-1) and β-catenin were investigated, and the results showed that a significant reduction in ZO-1 expression was observed in the tight junction of C14 seminiferous tubules (Figure 1h). In addition, the expression levels of β-catenin were greatly decreased in the spermatogonia and Sertoli cells of C14 compared to C13. ZO-1 and β-catenin were co-localized between Sertoli cells. The results suggest that BTB was disrupted in the C14 testes.

Taken together, it appears that the infertility of C14 may be due to the disruption of germ cell–Sertoli cell interactions, resulting in the adverse effects of sperm production by the failure of spermatogenesis.

### 2.2. Global Concentrations of 8-oxoGs in Sperm DNA

**Figure 2:**
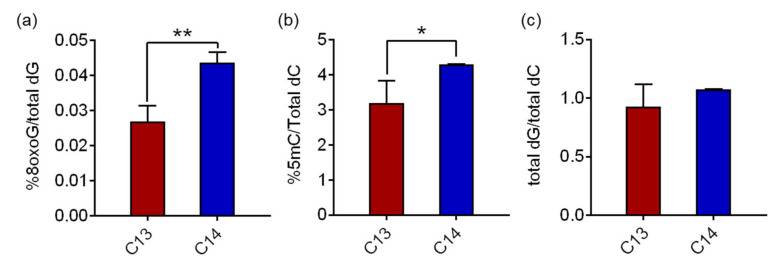
Significant increased levels of 8-oxoG in C14 sperm DNA. (**a**) Comparison of 8-oxoG levels in C13 and C14 sperm DNA. Total dG is the sum of 8-oxoG and dG. (**b**) Comparison of 5mC in C13 and C14 sperm DNA. Total dC is the sum of 5mC and dC. (**c**) The ratio of total dG and total dC. Bars indicate the average value of technical triplicate ± SD. * *p* < 0.05, ** *p* < 0.01 vs. C13.

Hydrolyzed genomic DNA samples from C13 and C14 sperm were analyzed using reverse-phase liquid chromatography/mass spectrometry (RP-LC/MS) to determine the 8-oxoG levels in the sperm DNA. The average 8-oxoG levels with standard deviation (SD) in C13 and C14 sperm were quantified as 0.027% ± 0.004% and 0.044% ± 0.003% of the total deoxyguanine (dG), respectively (Figure 2a). The 8-oxoG level of C14 sperm was 1.62 times higher than that of C13 with a significant difference (*p* = 0.0028). The global concentrations of 8-oxoG in sperm DNA were comparable to the historical range of 8-oxoG levels in pig livers, HeLa cells, calf thymus DNA, and our previous findings in mouse adipose tissue (ranged from 0.0214% to 0.0441%), even though the cell/tissue types were different. The 5-methylcytosine (5mC) levels in sperm, a canonical epigenetic marker in the genome, also changed consistently to 8-oxoG. The average 5mC levels in C13 and C14 sperm were 3.19% ± 0.641% and 4.30% ± 0.006% of the total deoxycytosine (dC), respectively, and the 5mC level of C14 sperm was 1.35 times higher than that of C13 with a significant difference (*p* = 0.048, Figure 2b). The quantity of the total dG and total dC was comparatively measured by high performance liquid chromatography (HPLC) for quality assurance of the procedure. In C13 and C14 sperm DNA, the ratios of total dG to total dC were 0.93 and 1.08, respectively, and there was no statistical difference, suggesting that there was no detection bias in this analysis.

These results demonstrate that 8-oxoG is present in fertile and infertile bull sperm at low but substantially different levels, and, in particular, is significantly higher in infertile sperm DNA. Therefore, in subsequent experiments, we investigated the possible role of increased 8-oxoG levels in spermatogenesis and, eventually, sperm fertility.

### 2.3. Distribution of 8-oxoG in Sperm DNA

We investigated the genomic distribution of 8-oxoG in the sperm DNA through next-generation sequencing (OG-seq). OG-seq produced for more than 84 billion paired-end reads with 96.56% ≥ Q30. From the input control DNA of C13 and C14 sperm DNA, 77.47% and 65.49% of the reads were successfully mapped on the bovine reference genome, respectively. In contrast, only 7.68% and 9.95% of the immunoprecipitated reads against 8-oxoG were mapped in C13 and C14 sperm DNA, respectively (Table 1). In particular, when comparing the mapping ratio (sample/input), that of C14 increased 1.5 times compared to C13, which was comparable to the quantitative result of 8-oxoG for each sperm DNA, using RP-LC/MS. OG-seq showed that there were 370 loci containing 8-oxoG in the C13 sperm DNA (Supplementary Table S1), and 614 loci in C14 sperm DNA (Supplementary Table S2).

**Figure 3:**
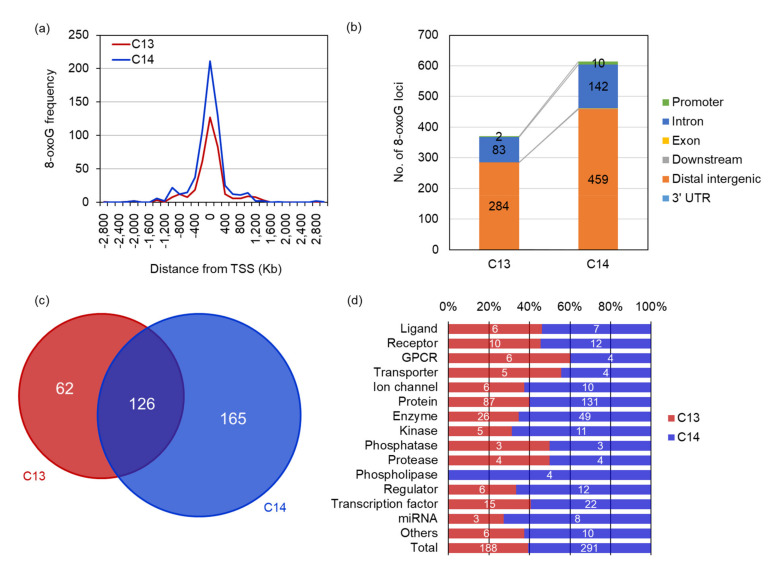
Increased number of 8-oxoG loci in C14 sperm DNA. (**a**) Distance of 8-oxoG loci from transcription start site (TSS). The vertical axis indicates the frequency of 8-oxoG loci for each bin of genomic distance from TSS. The red line represents C13 sperm and the blue line for C14 sperm. (**b**) Comparative genomic distribution of 8-oxoG loci of C13 and C14 sperm. Each color in the stacked graph represents from promoter (green) to 3′ UTR region (light blue). Numbers in the graph indicate the number of 8-oxoG loci in each genomic element. (**c**) Venn diagram of sperm genes harboring 8-oxoG. The red circle indicates the number of C13 sperm genes containing 8-oxoG and the blue circle for C14 sperm genes. (**d**) Protein annotation encoded by 8-oxoG genes. The red bar represents the C13 sperm proteins and the blue bar for the C14 sperm proteins. The number in the bar represents the number of the protein encoded by 8-oxoG genes. The ratio of the total 8-oxoG genes of C13 and C14 sperm is shown below.

The genomic distribution analysis of the 8-oxoG loci revealed that most of them were distributed around the transcription start site (TSS) and also in the distal intergenic and intron regions. Approximately 81.6% and 82.9% of the 8-oxoG in C13 and C14 sperm DNA were distributed within TSS ± 400 Kb, respectively, and there was no difference in the 8-oxoG distribution between them (Figure 3a). The distal intergenic and intron regions had 99.2% and 97.9% of 8-oxoG, respectively, and no difference was observed between C13 and C14. However, only two promoter regions with 8-oxoG were found in C13 sperm DNA, whereas 10 promoter regions were observed in C14 sperm DNA, and the distribution of 8-oxoG in the C14 promoter regions increased by five times compared to C13 promoters (Figure 3b). Annotation of the genomic loci containing 8-oxoG showed that 188 and 291 genes harbored 8-oxoG in their genetic elements (8-oxoG genes). Among them, 126 genes were common in both C13 and C14 sperm DNA, and 62 and 165 genes were exclusively annotated in C13 and C14 sperm, respectively (Figure 3c). The annotated products encoded by 8-oxoG genes showed that various kinds of proteins had 8-oxoG in their genomic elements, including the ligand and transcription factor. In most protein classes, no frequency difference was observed between C13 and C14 sperm in the content of 8-oxoG. However, the number of 8-oxoG genes encoding kinases, phospholipase, regulators, and miRNA were more than doubled in C14 sperm compared to C13, which exceeded the total ratio of 8-oxoG genes between C13 and C14 sperm. In particular, genes encoding phospholipase was observed only in the C14 sperm (Figure 3d).

These results suggest that 8-oxoG may play a role in regulating sperm gene activity and that there may have been a problem with acrosomal exocytosis through phospholipase dysregulation during the spermatogenesis of C14 sperm.

### 2.4. Functional Enrichment of 8-oxoG Genes of Sperm

**Figure 4:**
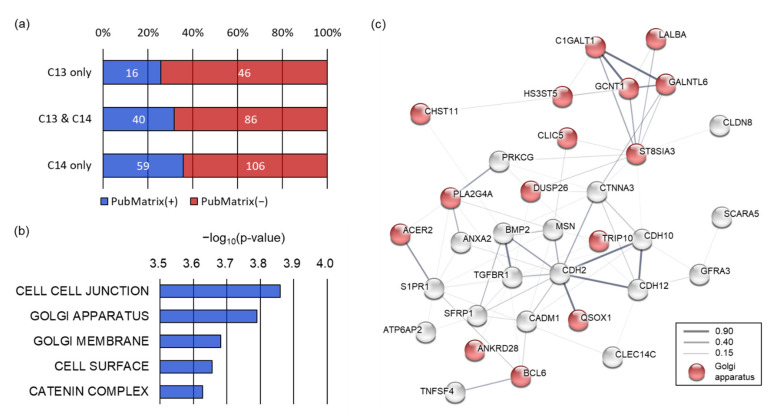
Golgi component affected by 8-oxoG in C14 sperm DNA. (**a**) Literature association of sperm genes containing 8-oxoG. Blue bars indicate the number of 8-oxoG genes with literature related to sperm (PubMatrix (+)) and red bars for genes without sperm-related literature (PubMatrix (−)). The number in the bar represents the number of literatures related to sperm. (**b**) GSEA analysis of 8-oxoG genes exclusive to C14 sperm. Horizontal axis indicates (−)log_10_-transformed *p*-value of each cellular component terminology. (**c**) Protein–protein interaction network analysis of functionally enriched 8-oxoG genes of C14 sperm. Nodes indicate the cellular component-enriched genes. Edge thickness indicates the interaction score. Red nodes represent the Golgi apparatus-enriched genes.

To understand how genes containing 8-oxoG correlate with the sperm phenotype, we performed literature mining. Multiple text-based mining demonstrated that 16 out of 62 8-oxoG genes (25.8%) exclusive to C13 sperm were associated with sperm terms (spermatogenesis, spermiogenesis, asthenozoospermia, or teratozoospermia). In contrast, 59 of the 165 8-oxoG genes (35.8%) exclusive to C14 sperm were observed to be associated with the sperm terms, and the literature count increased marginally for C14 sperm compared to C13 sperm (*p* = 0.0778) (Figure 4a). We then performed a gene set enrichment analysis (GSEA) using 165 8-oxoG genes exclusive to C14 sperm to understand the function of the 8-oxoG genes in the infertile sperm.

GSEA with a biological process collection demonstrated that the 72 genes out of 8-oxoG genes in C14 sperm were related to sperm motility, including cell projection organization (*p* = 4.6 × 10^−5^), cell motility (*p* = 8.29 × 10^−5^), and negative regulation of cell–cell adhesion (*p* = 1.05 × 10^−4^). These biological processes were significantly inter-connected with each other (enrichment *p* < 0.01) (Supplementary Figure S1). Therefore, these results would explain the genetic basis of asthenozoospermia of the C14 bull. GSEA with a cellular component collection revealed that the 8-oxoG genes of C14 sperm were significantly enriched in cell–cell junctions (*p* = 0.000138), the Golgi apparatus (*p* = 0.000162), the Golgi membrane (*p* = 0.000208), the cell surface (*p* = 0.000221), and the catenin complex (*p* = 0.000236) (Figure 4b). Protein–protein interaction network analysis showed that the 8-oxoG genes of C14 sperm enriched in the cellular components were well interconnected with each other (interaction score ≥ 0.15) based on various interaction sources, including text-mining, experiments, databases, co-expression, neighborhood, gene fusion, and co-occurrence. In this network, 16 of the 8-oxoG genes exclusive C14 sperm were significantly enriched in the Golgi apparatus (false discovery rate *q* = 2.2 × 10^−7^).

The Golgi apparatus has been known to be an important sperm-specific intracellular organelle essential for the acrosome formation; therefore, these results imply that 8-oxoG occurring in genes participating in the Golgi apparatus may have caused abnormalities in the spermiogenesis and sperm fertility in C14.

### 2.5. Differentially Expressed Proteins (DEPs) in C14 Sperm

**Figure 5:**
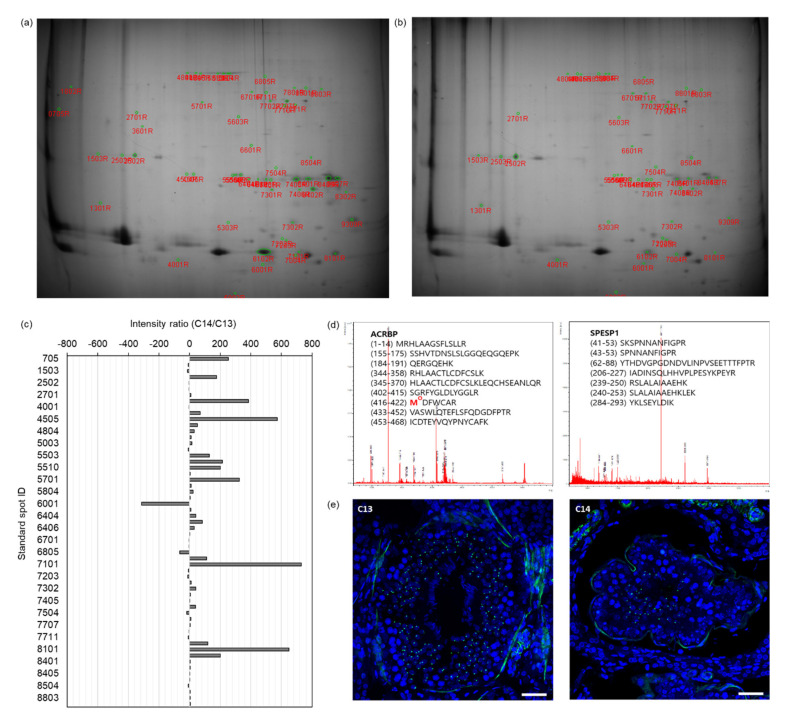
Differentially expressed proteins (DEPs) in C14 sperm. (**a**) Separation of C13 sperm proteins by 2-dimensional polyacrylamide gel electrophoresis (2D-PAGE). (**b**) Separation of C14 sperm proteins by 2D-PAGE. (**c**) Intensity ratio of the selected standard spots between C13 and C14 sperm proteins. The horizontal axis indicates the intensity ratio of C14 over C13 and the vertical axis for standard spot ID. (**d**) Representative peptide mass fingerprinting (PMF) spectra of down-regulated ACRBP and SPESP1 proteins in C14 sperm. The numbers in parentheses indicate the start to end of the amino acid residue of the peptide, followed by the amino acid sequence of the peptide. M^o^ indicates the oxidized methionine. (**e**) Immunolocalization of ACRBP in the testes. Green spots indicate ACRBP in the secondary spermatocytes and round spermatids. Scale bar represents 50 µm.

To verify the abnormality of acrosome formation predicted through bioinformatics analysis of the OG-seq data from C14 sperm DNA, we performed proteomic analysis of C14 sperm proteins. The proteins from C13 and C14 sperm were separated by isoelectric focusing followed by SDS-PAGE (Figure 4a,b). Based on the spot intensity, we identified 58 differentially expressed standard spots in C14 sperm proteins with at least a two-times greater intensity difference (Figure 4c). The fold change of the spot intensity ranged from a 313-fold decrease to a 731-fold increase in the C14 sperm proteins compared to the C13 sperm proteins (Supplementary Table S3). Among them, we conducted peptide mass fingerprinting (PMF) by selecting the top 19 spots with the highest fold change and identified 10 DEPs in C14 sperm (protein probability ≤ 10^−92^, *p* ≤ 0.0032). Representative PMF spectra and the identified matched amino acid sequence are presented in Figure 5d.

In the DEPs, the expression levels of six proteins were upregulated in C14 sperm compared with C13 sperm, including annexin A4 (ANXA4, fold increase ≥ 128.0), ubiquinol–cytochrome C reductase core protein 1 (UQCRC1, fold increase = 326.4), tubulin alpha-3 chain-like (LOC10184899, fold increase = 176.0), succinyl-CoA:3-ketoacid-coenzyme A transferase 2, mitochondrial (OXCT2, fold increase = 119.4), phosphatidylethanolamine-binding protein 4 (PEBP4, fold increase = 68.7), and serum albumin (ALB, fold increase ≥ 23.7). In contrast, the expression levels of four proteins, ACRBP (fold decrease ≤1.6), outer dense fiber protein 2 (ODF2, fold decrease ≤1.6), SPESP1 (fold decrease ≤2.6), and Izumo sperm–egg fusion protein 4 (IZUMO4, fold decrease = 9.8), were downregulated in C14 sperm compared to C13 sperm (Table 2).

The IF assay verified the differential expression of ACRBP during spermatogenesis (Figure 5e). In the seminiferous tubes of the testes of C13 and C14, differentiating sperm cells corresponding to the entire stages of spermatogenesis were observed, including those from spermatogonia to the elongated spermatid. ACRBP signals were observed in the nucleus of secondary spermatocytes and round spermatids but were absent in spermatogonia, primary spermatocytes, and elongated spermatids in both testes. However, a significant reduction in ACRBP-positive cells was observed in the testes of C14 compared to those in C13.

The DEPs identified through proteomics analysis did not exactly match with the 8-oxoG genes identified by OG-seq. However, the DEPs of C14 sperm, including ACRBP and SPESP1, suggest abnormal acrosome formation, which was consistent with the results of the functional enrichment analysis of the 8-oxoG genes of C14 sperm. Therefore, these results suggest that 8-oxoG formation during spermatogenesis dysregulated the activity of the acrosome-related genes, leading to the production of sperm with structural and functional defects in the C14 bull, resulting in infertility.

## 3. Discussion

In livestock, inbreeding is considered to depress genetic gain and is particularly pointed out as a cause of the decline in the reproductive capacity of males [13]. However, the exact cause of male infertility is not fully understood, and sperm infertility, like asthenoteratozoospermia, cannot be restored by nutritional or medicinal treatment [14,15]. Therefore, identifying the direct cause of infertile sperm is considered the first step in finding molecular markers to estimate male infertility. In this study, we found an infertile bull with asthenoteratozoospermia, which had a significantly higher level of 8-oxoG formation in its sperm DNA. Histological analysis of the testes of the infertile bull revealed abnormal phenotypes of the seminiferous tubules, including disorganization of germ cells, dramatic reduction of spermatogonia, spermatocytes, spermatids, and spermatozoa during the process of spermatogenesis. Here, we provide evidence that these abnormalities were potentially caused by functional failure of the Sertoli cells that play a critical role in the regulation of spermatogenesis. In addition, the expression of ZO-1 and β-catenin was dramatically reduced in the seminiferous tubules. ZO-1, tight junction protein (TJP), and β-catenin, adhesion junction protein (AJP), are required for the junctions between Sertoli cells and between Sertoli cells and germ cells, respectively [16]. Thus, impaired Sertoli cell function may be the primary cause of the disruption of TJP and AJP involved in the formation of the blood–testis barrier (BTB). Therefore, it appears that defects in BTB due to excess ROS may cause microenvironmental homeostasis failure for spermatogenesis in the seminiferous tubules.

The acrosome is a unique structure of the sperm head of many mammalian species and highly conserved throughout evolution. During spermatogenesis in testes, the Golgi apparatus of spermatid cells packs the center of the acrosome capsule, which contains many different enzymes. The spermatid cells use the proteins in the endoplasmic reticulum and Golgi network to accumulate hydrolytic enzymes into the acrosome center. Therefore, Golgi-derived biosynthetic cargo destined for the acrosome is essential for normal acrosome formation. The primary function of the acrosome is the acrosomal reaction that induces fusion between the sperm and oocyte during fertilization. The acrosome also has a role in sperm morphogenesis [17]. Acrosomal vesicles are produced in the area around the nucleus through interaction with the Golgi apparatus in the first stage of spermatogenesis, and remodeling of the vesicle in the late stage, forming the characteristic shape of the sperm head. Subsequently, the sperm that move to the epididymis undergo maturation to complete acrosome formation [18,19,20]. The unique structure of the acrosome, which is composed of membranes and proteins, is especially susceptible to ROS [21]. The acrosomal structure and function are adversely influenced by protein oxidation and lipid peroxidation in the acrosomal membrane caused by ROS [22,23]. Along with the reduced ability to repair DNA damage during spermiogenesis, the vulnerability of the acrosome to ROS has been suggested as one of the major causes of male infertility. However, the molecular genetic mechanisms for reduced spermatogenesis and sperm fertility by ROS are unclear. In this study, we observed infertility in one of the 35 bulls raised under the same breeding conditions due to complete asthenoteratozoospermia for unexplained reasons. Through the sperm analysis of this infertile bull, a significantly high level of 8-oxoG formation was observed in the sperm DNA, and these oxidative lesions were particularly located around the TSS and genes involved in the Golgi apparatus. Eventually the formation of the acrosome appeared to be affected. To our knowledge, this is the first time that 8-oxoG formation due to oxidative stress has been investigated at the genomic level in sperm. In particular, it is of high relevance to suggest a molecular mechanism of action of 8-oxoG lesions on spermatogenesis and sperm fertility.

DNA damage is associated with the impairment of DNA protamination during late spermatogenesis, and a significant increase in the 8-oxoG levels is known to disrupt the chromatin remodeling and ultimately lead to DNA fragmentation [24]. However, except for structural defects in DNA, no intensive analysis of the genetic network related to spermatogenesis and selectivity of the genomic loci where 8-oxoG is generated has yet been reported. In this study, we identified that 165 genes of infertile sperm had 8-oxoG lesions, and 15 of them were genes possibly involved in the formation of the Golgi apparatus. Literature mining revealed that five of the Golgi-associated genes play major roles in spermatogenesis, sperm motility, and sperm fertility. For example, B-cell CLL/lymphoma 6 (BLC6) was reported to act as a stabilizer to protect sperm from apoptosis by various stressors [25], and quiescin sulfhydryl oxidase 1 (QSOX1) was reported to acquire fertility and the structural stability of sperm through sulfhydryl oxidation [26,27]. Phospholipase A2 (PLA2) is known to be involved in the biosynthesis of eicosanoids as an indicator of oxidative stress in various infertile conditions, and the active form of PLA2 is present prominently in the head of impaired motile sperm [28,29,30]. Chloride intracellular channel 5 (CLIC5) is also known to play a role in acquiring sperm motility through interactions with protein phosphatase 1 (PP1) [31]. Research has yet to determine how the activity of these genes is regulated by 8-oxoG lesions caused by oxidative stress. Considering the regulation of gene activity through the deletion of the corresponding genomic loci by 8-oxoG or the epigenetic function of 8-oxoG [32,33,34], subsequent studies on these subjects would be valuable for a genetic understanding of the impairment of spermatogenesis caused by oxidative stress.

While this study provides an important basis for understanding the genetic mechanism of 8-oxoG-induced defects in spermatogenesis, there are several limitations. First, the 8-oxoG level in the sperm DNA was higher than in previous studies [35,36]. This discrepancy is likely mainly due to differences in the samples and analytical equipment. The higher 8-oxoG levels observed in this study would also come from the artificial factor during sample preparation [37]. However, an appropriate reducing agent was applied during sample preparation, and the 8-oxoG levels were consistently observed when analyzed by two independent LC/MS and OG-seq methods. Therefore, we emphasize that the 8-oxoG found was native instead of artificial. Secondly, it is not possible to obtain sufficient statistical power by analyzing sperm obtained from one fertile bull and one infertile bull. Severe sperm abnormalities are rare events in mammals. From 2012 to 2018, a total of 34 bulls were bred at our farm facility and only one bull, C14, was observed to have asthenoteratozoospermia with infertility. Despite this shortage of sample numbers, we conducted comprehensive multidisciplinary studies of the sperm and the male reproductive tract. The conclusions drawn from this analysis would not, therefore, be appropriate to extend to bulls and mammals as a whole; however, their meaning could be acknowledged. Finally, in the comparison between the genome and the proteome obtained from the infertile sperm, we did not observe a correlation between the 8-oxoG genes and their encoded proteins. Instead, the 8-oxoG genes and DEPs observed in infertile sperm exhibited a common defect of acrosome formation that can occur in late spermatogenesis. This is likely due to the fact that the change in gene activity by 8-oxoG occurs during the spermatogenic process, and the DNA and proteome of the sperm we analyzed contains only traces of abnormalities in the gene network related to spermiogenesis.

In conclusion, we found a high distribution of 8-oxoG in sperm DNA that lost fertility due to asthenoteratozoospermia. The majority of the 8-oxoG loci were distributed across the TSS, and 8-oxoG was found to disrupt genes linked to the formation of acrosomes originating from the Golgi apparatus. Proteomic analysis confirmed that acrosome-associated proteins were downregulated in infertile sperm. This preliminary case report with advanced techniques suggests that 8-oxoG formation during spermatogenesis may lead to structural and functional defects in sperm by failing to regulate the acrosome-related gene activity. These findings may provide new insights to understand bovine male infertility.

## 4. Materials and Methods

### 4.1. Animals

Two Korean native striped bulls at 6 years old (C13 and C14) were chosen for this study because C13 was observed to have normal reproductive activity while C14 was infertile. The semen samples of these bulls were washed with phosphate buffered solution (PBS; Thermo Fisher Scientific, Waltham, MA, USA) and cryopreserved until use. After the cryopreservation of semen, the testes and epididymis of the bulls were collected by castration. All procedures described were reviewed and approved by the Institutional Animal Care and Use Committee at the National Institute of Animal Science (Approval No. NIAS 2014–489).

### 4.2. Sperm Analysis

A volume of 5 mL semen was diluted with 45 mL of triladyl solution (Minitüb GmbH, Tiefenbach, Germany) and mixed on a ThermoMixer (Eppendorf, Hamburg, Germany) with a mixing frequency of 300 rpm at 34.0 °C. After 30 min of mixing, the final concentration of sperm was adjusted to 80 × 10^6^ sperm/mL, using a Makler counting chamber (Sefi-Medical Instruments, Haifa, Israel). The motility and progressiveness of sperm were analyzed using a microscope equipped with a computer-assisted sperm analyzer (CASA; Proiser, Paterna, Spain) with at least 200 sperm per bull. To analyze the sperm morphology, more than 200 sperm per bull were smeared on a glass slide (Paul Marienfeld GmbH & Co.KG, Lauda-Königshofen, Germany) and stained, using the Differential Quik III stain kit (Polysciences, Warrington, PA, USA). The stained slide was allowed to air dry and observed with a bright field microscope (Olympus, Tokyo, Japan) according to the criteria of sperm morphology [38].

### 4.3. Acrosome Analysis

Thawed semen was mixed with 10 mL PBS (Thermo Fisher Scientific) and centrifuged at 300 *g* for 10 min. After removing some of the supernatant, the sperm pellets were resuspended by gentle tapping. The sperm suspension of around 10 µL was smeared on the slide glass (Paul Marienfeld GmbH & Co.KG), dried, and fixed for 5 min in PBS containing 3.7% (*w*/*v*) paraformaldehyde (Sigma-Aldrich, St. Louis, MO, USA). The fixed sperm smear was stained with 0.2% (*w*/*v*) Coomassie Brilliant Blue (CBB) solution (Sigma-Aldrich) for 2 min and transferred to distilled water for 5 s. After drying the slide on a slide warmer (Changshin Science, Seoul, Korea) at 37 °C for 2 min, the protein intensity (*n* = 25 per bull) and the integrity of the acrosome in the sperm head (technical triplicates, *n* = 200 per bull) were evaluated under a microscope (Olympus, Tokyo, Japan). The acrosome protein intensity was quantified by using ImageJ (version 1.38e) [39].

### 4.4. Histological and Immunofluorescence (IF) Analysis of Male Reproductive Tract

The testicular tissues were fixed in 4% (*v*/*v*) paraformaldehyde (Thermo Fisher Scientific) in PBS for 24 h at room temperature. After rinsing, the tissues were cryoprotected by incubating in 30% (*w*/*v*) sucrose (Thermo Fisher Scientific) in PBS for 24 h. The tissues were embedded in Tissue-Tek compound (Sakura Finetek, Torrance, CA, USA), mounted on a cutting block, and frozen. The tissue was then cut at 10 µm of thickness, using a Leica 3050 cryostat (Leica Microsystems, Bannockburn, IL, USA). The sections were placed onto Fisher Superfrost Plus microscope slides (Fisher Scientific, Pittsburgh, PA, USA) and stored at −20 °C until use. For histological analysis, hematoxylin and eosin (H&E) staining was performed as described before [40]. For IF analysis, the sections were hydrated in PBS for 10 min, and heated by microwaving in an alkaline buffer (Vector Laboratory, Burlingame, CA, USA) three times for 2 min each with 5 min intervals for antigen retrieval. After blocking with 1% (*w*/*v*) bovine serum albumin (Thermo Fisher Scientific) in PBS for 60 min at room temperature, the sections were incubated with primary antibodies in a moist chamber for 90 min at room temperature or overnight at 4 °C, and then incubated with secondary antibodies for 60 min at room temperature. Detailed information on the primary and the secondary antibodies are provided in Supplementary Table S4. Confocal images were acquired using a confocal microscope and analyzed using Zen Blue software (Zeiss, Oberkochen, Germany).

### 4.5. Reverse Phase-Liquid Chromatography/Mass Spectrometry (RP-LC/MS) Measurement of 8-oxoG

Genomic DNA was extracted from sperm, using the DNeasy Blood and Tissue kit (Qiagen, Valencia, CA, USA) with supplementation of desferal and butylated hydroxytoluene at 100 µM each (Sigma-Aldrich, St. Louis, MO, USA). The extracted DNA was enzymatically hydrolyzed as previously described [34]. 8-[15N5]oxoG at 10 nM was used as a spike control (Cambridge Isotope Laboratories, Inc., Tewksbury, MA, USA).

For quantification of 8-oxoG, RP-LC/MS consisting of an Ultimate 3000 RS high performance liquid chromatography (HPLC) instrument (Dionex, Sunnyvale, CA, USA) equipped with a TSQ ENDURA triple-quadrupole mass spectrometer (Thermo Fisher Scientific) and a reversed-phase chromatography column (2.1 × 100 mm, inner diameter = 2.6 µm, Phenomenex, Torrance, CA, USA) was used as previously described [34]. Separate samples were subjected to quantify dG and dC levels by using high-performance liquid chromatography (HPLC) equipped with the Shiseido column (Capcell Pak C18 UG120 4.6 × 250 mm, inner diameter = 5 µm, Phenomenex).

### 4.6. 8-oxoG Enrichment by Affinity Purification

DNA fragments with 8-oxoG were purified as previously described [41]. Briefly, 5 µg of the genomic DNA extracted from sperm was fragmented with an S2 ultrasonicator (Covaris, Woburn, MA, USA) in 10 mM Tris buffer (pH 8.0) to obtain approximately 150-bp fragments. After sonication, the fragmented DNA was concentrated to 20 µL in 100 mM NaPi buffer (pH 8.0), using QIAquick PCR purification kit (Qiagen). A 100 µL volume of 100 mM NaPi buffer with 20 mM amine-PEG2-biotin (Thermo Fisher Scientific) was added and the mixture was heated to 75 °C for 10 min. After thermal equilibration to room temperature, 5 mM K_2_IrBr_6_ (Sigma-Aldrich) was added for 8-oxoG biotinylation. The biotinylated DNA fragments were eluted with 125 µL Tris buffer using the QIAquick PCR purification kit and collected using Dynabeads MyOne Streptavidin C1 (Thermo Fisher Scientific). Complementary DNA strands to those with bound biotinylated 8-oxoG were released by incubation in 150 mM NaOH at 20 °C for 30 min and concentrated to 10 µL ddH_2_O using the ssDNA/RNA Clean & Concentrator kit (Zymo Research, Irvine, CA, USA).

### 4.7. 8-oxoG Sequencing (OG-Seq)

The 8-oxoG-enriched DNA fragments were applied for next-generation sequencing. The OG-seq library was constructed using the TruSeq Nano DNA kit (Illumina, San Diego, CA, USA) and sequenced with TruSeq SBS Kit v3-HS on a HiSeq 2000 sequencer (Illumina) to obtain 101-bp paired-end reads. Image analysis and base calls were performed using the Illumina pipeline (v1.8) with default settings. Reads were aligned to the bovine reference genome (bosTau8) using the Isaac aligner (Illumina). Duplicate reads were identified and removed as Picard (http://broadinstitute.github.io/picard, version 2.25.5, access date 6 December 2018). In the reads mapped using MACS241, the enhanced peaks were called, and the peaks were annotated with ChIPseeker. 8-OxoG loci that differed significantly from the input DNA (fold change >2, *q* < 0.05) were selected for further analysis.

### 4.8. Bioinformatic Analyses

The biological relevance of genes harboring 8-oxoG loci (8-oxoG genes) was investigated using PubMatrix, a text-based data mining tool [42]. Search terms (8-oxoG gene symbols) and modified terms (sperm, spermatogenesis, spermiogenesis, asthenozoospermia, and teratozoospermia) were used for pair-wise comparison.

8-OxoG gene lists from C13 and C14 sperm DNA were compared with Venn diagrams (https://bioinfogp.cnb.csic.es/tools/venny, version 2.1, access date 13 Jan 2021), and C14 exclusive genes were subjected to gene set enrichment analysis (GSEA) [43] to determine the biological process and cellular component that they participated in, using a false discovery rate (FDR) q-value cutoff of 0.05.

A regulatory network was constructed, using the STRING protein–protein association network database (https://string-db.org, version 11.0, access date 13 January 2021) [44]. C14 sperm-specific 8-oxoG genes were used with more than 0.15 of interaction confidence.

### 4.9. 2-Dimensional Polyacrylamide Gel Electrophoresis (2D-PAGE)

Sperm pellets were homogenized in a lysis buffer composed of 7 M urea, 2 M thiourea, 4% (*w*/*v*) CHAPS, 1% (*w*/*v*) dithiothreitol, 1 mM benzamidine (Sigma-Aldrich), and 2% (*v*/*v*) pharmalyte (Amersham Biosciences, Little Chalfont, U.K.). Proteins were extracted by vortexing for 1 h. After centrifugation at 15,000× *g* for 1 h at 15 °C, soluble fraction was applied to 2D-PAGE.

Proteins were applied to pre-equilibrated IPG dry strips (Genomine, Gyeongsangbuk-do, South Korea) and isoelectric focusing (IEF) was performed at 20 °C, using a Multiphor II electrophoresis unit (Amersham Biosciences). After IEF, 10–16% (*w*/*v*) SDS-PAGE was performed, using the Hoefer DALT 2D system (Amersham Biosciences), and the 2D gel was stained with Coomassie blue. Quantitative analysis of the digitized images was carried out using the PDQuest (BioRad, Hercules, CA, USA) and the quantity of each spot was normalized by total valid spot intensity.

### 4.10. Peptide Mass Fingerprinting (PMF)

For protein identification, protein spots were excised and digested with trypsin (Promega, Madison, WI, USA). The digested peptides were mixed with α-cyano-4-hydroxycinnamic acid in 50% (*v*/*v*) acetonitrile in 0.1% trifluoroacetic acid and subjected to matrix-assisted laser desorption ionization-time-of-flight (MALDI-TOF) analysis (Bruker Daltonics, Billerica, MA, USA) as previously described [45]. Spectra were collected from 300 shots per spectrum between m/z ranged from 600 to 3000 and calibrated by using trypsin auto-digestion peaks (m/z = 842.5099, 2211.1046). The peak list was generated using Flex Analysis 3.0 (Bruker Daltonics). MASCOT (Matrix Science Inc., Boston, MA, USA) was used for protein identification by PMF, using the following parameters: trypsin as the cleaving enzyme, a maximum of one missed cleavage, iodoacetamide on cysteine (Cys) as a complete modification, oxidation on methionine (Met) as a partial modification, monoisotopic masses, and a mass tolerance of ±0.1 Da.

### 4.11. Statistical Analysis

Data obtained from the sperm of both the fertile and infertile bull were measured in technical triplicate and then were subjected to statistical analysis. Those included sperm indexes (sperm motility, sperm progressiveness, acrosome analyses) and chromatographic analysis of the sperm DNA (8-oxoG, 5 mC).

All data were analyzed with Student *t*-tests or Mann–Whitney U tests, depending on the homogeneity of the variance of the data. All statistical analyses were performed using Prism software (GraphPad Software Inc., San Diego, CA, USA), with *p*-values < 0.05 considered to indicate statistical significance. All measurements are reported as means ± standard deviation (SD).

## Figures and Tables

**Table 1:** Statistics of 8-oxoG sequencing (OG-seq) data.

Sample	Total Read Bases	Total Reads	GC (%)	Q20 (%)	Q30 (%)	Mapping Ratio (%)
C13 input	9,847,220,343	97,606,476	42.39	98.30	97.04	77.47
C13	8,540,190,885	84,978,688	43.51	98.39	97.21	7.68
C14 input	10,909,682,941	108,190,516	44.99	98.64	97.61	65.49
C14	10,207,200,528	101,407,472	43.15	98.01	96.56	9.95

Total read bases: Total number of read bases (total reads x read length) after trimming. Total reads: Total number of reads after trimming. GC (%): GC Content. Q20 (%): Ratio of reads that have phred quality score over 20. Q30 (%): Ratio of reads that have phred quality score over 30.

**Table 2:** Differentially expressed proteins (DEPs) in C14 sperm compared to C13 sperm.

SSP	MW	PI	Abundance		Fold Change (C14/C13)	Protein Name	Protein Score	Expected *p*-Value
			C13	C14				
1503	24.02	4.48	1005.9	71.0	−14.2	Acrosin-binding protein	130	4.5 × 10^−7^
1802	50.63	4.23	1.0	176.0	176.0	Tubulin alpha-3 chain-like	137	9.1 × 10^−8^
2502	23.73	4.83	5822.0	3749.4	−1.6	Acrosin-binding protein	111	3.6 × 10^−5^
2503	23.80	4.70	1564.3	510.2	−3.1	Acrosin-binding protein	107	9.1 × 10^−5^
4504	20.52	5.32	1.0	68.7	68.7	Phosphatidylethanolamine-binding protein 4	94	1.6 × 10^−3^
4505	20.49	5.38	1.0	573.6	573.6	Annexin A4 isoform 3	116	1.1 × 10^−5^
4801	60.74	5.32	8.8	447.3	50.7	Serum albumin	160	4.5 × 10^−10^
4804	60.65	5.40	17.3	502.2	29.0	Serum albumin	252	2.9 × 10^−19^
5503	20.41	6.11	18.0	2301.0	128.0	Annexin A4	120	4.5 × 10^−6^
5508	20.39	6.00	10.2	2177.5	213.1	Annexin A4	117	9.1 × 10^−6^
5510	20.37	5.94	3.1	623.0	200.3	Annexin A4	121	3.6 × 10^−6^
5701	39.25	5.46	1.0	326.4	326.4	Ubiquinol–cytochrome C reductase core protein 1	128	7.2 × 10^−7^
5804	60.48	5.85	97.2	2308.0	23.7	Serum albumin	279	5.7 × 10^−22^
6701	46.49	6.35	611.8	371.5	−1.6	Outer dense fiber protein 2 isoform X3	248	7.2 × 10^−19^
6711	46.31	6.67	540.7	142.0	−3.8	Outer dense fiber protein 2 isoform X14	126	1.1 × 10^−6^
7710	37.70	7.07	482.4	182.5	−2.6	Sperm equatorial segment protein 1	112	2.9 × 10^−5^
7711	38.14	7.31	282.2	28.8	−9.8	Sperm equatorial segment protein 1	111	3.6 × 10^−5^
7801	50.04	7.28	1.0	119.4	119.4	Succinyl-CoA:3-ketoacid-coenzyme A transferase 2, mitochondrial	92	3.2 × 10^−3^
8504	23.33	7.61	470.4	48.1	−9.8	Izumo sperm–egg fusion protein 4	95	1.3 × 10^−3^

Protein score = −10* Log(P), where P is the probability that the observed match is a random event.

## Data Availability

OG-seq raw and processed data from this study have been submitted to the NCBI Gene Expression Omnibus (GEO; http://www.ncbi.nlm.nih.gov/geo/, access date 25 January 2021) under accession number GSE165538.

## Supplementary Materials

Supplementary tables are available online at https://www.mdpi.com/article/10.3390/ijms22115857/s1.

## Abbreviations

2D-PAGE	2-dimensional polyacrylamide gel electrophoresis
8-oxoG	8-oxoguanine
ACRBP	Acrosin-binding protein
CASA	Computer-assisted sperm analysis
DEPs	Differentially expressed proteins
GSEA	Gene set enrichment analysis
OG-seq	Genomic distribution of 8-oxoG by next-generation sequencing
OXCT1	3-oxoacid CoA-Transferase 1
PMF	Peptide mass fingerprinting
RP-LC/MS	Reverse-phase liquid chromatography/mass spectrometry
SPESP1	Sperm equatorial segment protein 1
V-ATPase B1	Vacuolar-type ATPase subunit B1
ZO-1	Zonula occludens 1
