# Supplementary material for: Dysregulation of the Acrosome Formation Network by 8-oxoguanine (8-oxoG) in Infertile Sperm: A Case Report with Advanced Techniques
Source: Int J Mol Sci. 2021 May 30;22(11):5857. doi: 10.3390/ijms22115857 (PMC8199233; doi:10.3390/ijms22115857)

Dysregulation of the acrosome formation network by 8-oxoguanine (8-oxoG) in infertile sperm

Sung Woo Kim[1#], Bongki Kim[2#], Jongsoo Mok[3], Eun Seo Kim[3], Joonghoon Park[3,4*]

[1]Animal Genetic Resources Research Center, National Institute of Animal Science (NIAS), Rural Development Administration (RDA), Republic of Korea.

[2]Department of Animal Resources Science, Kongju National University, Republic of Korea.

[3]Department of International Agricultural Technology, Graduate School of International Agricultural Technology, Seoul National University, Republic of Korea

[4]Institute of GreenBio Science and Technology, Seoul National University, Republic of Korea

#These authors contributed equally to this work

*Author to whom correspondence should be addressed (joonghoon@snu.ac.kr)

Keywords: 8-oxoguanine (8-oxoG), Acrosome, Sperm, Infertility, Bull

Supplementary Tables

Supplementary Table 1. Annotation of 8-oxoG loci in C13 sperm DNA

Supplementary Table 2. Annotation of 8-oxoG loci in C14 sperm DNA

Supplementary Table 3. Intensity of differentially expressed standard spots of C14 sperm proteins

Supplementary Table 4. Antibody information

Supplementary Figures

Supplementary Figure 1. Functional enrichment of 8-oxoG genes exclusive to C14 sperm.

(a) Gene set enrichment analysis (GSEA) with biological process collection. Sperm motility-related biological processes were selected and depicted with (-)log-transformed p-value. (b) Biological process enrichment network. Sperm motility-related biological processes were depicted in differently colored terms. Each colored node represents each cluster of biological processes. The node size is proportional to the number of proteins belonging to the processes and the edge thickness to the numbers of proteins common in the processes.

Supplementary Figure 1

(a)

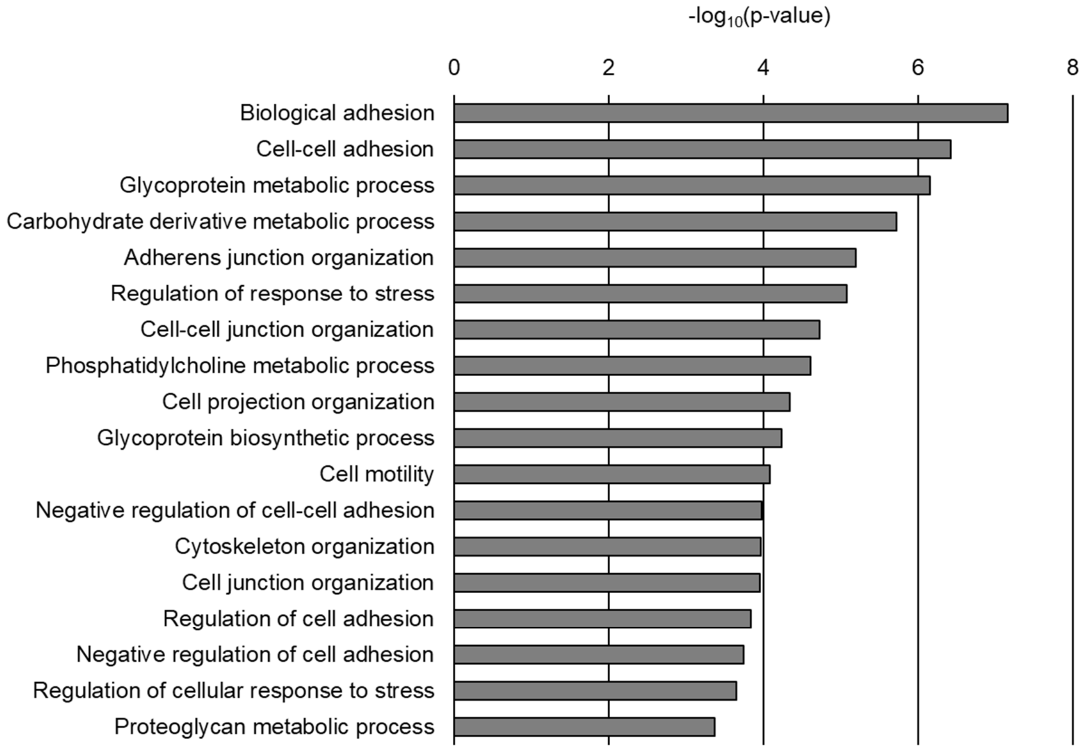

(b)

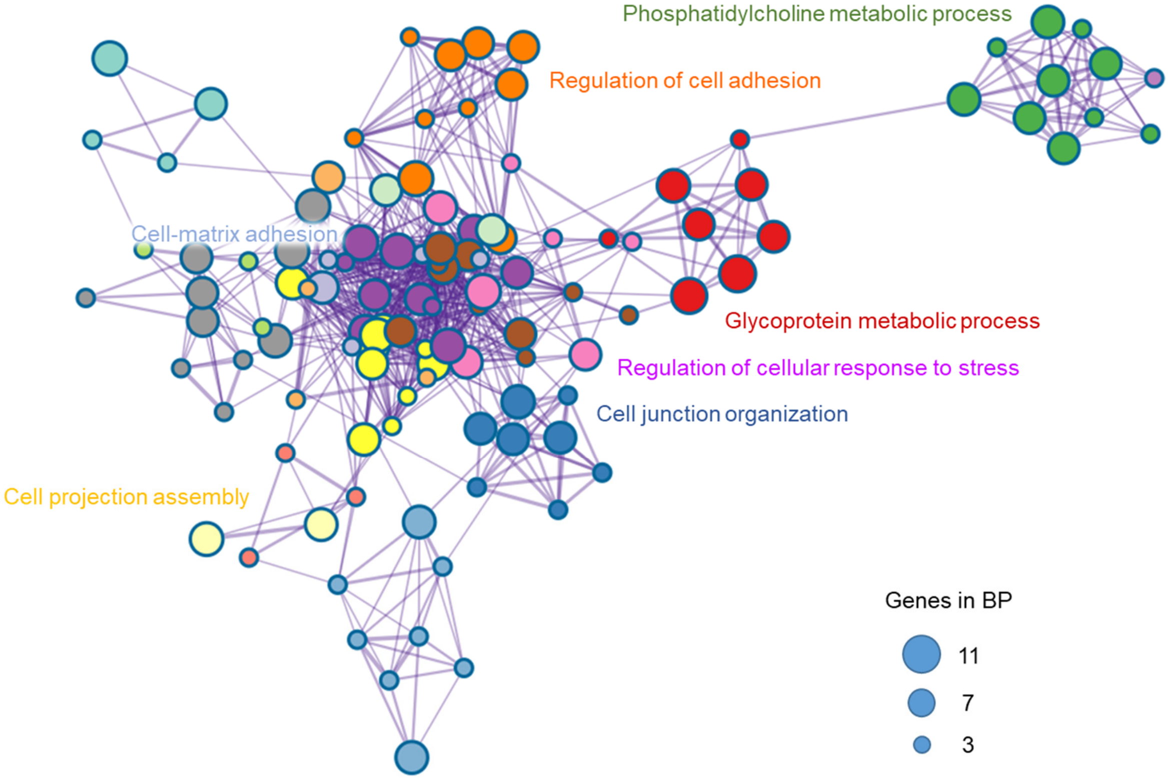

Supplement: Supplementary file 1 [file ijms-22-05857-s001.zip › ijms-1188060R1_Supple cover page.pdf]
