# Supplementary material for: Dysregulation of the Acrosome Formation Network by 8-oxoguanine (8-oxoG) in Infertile Sperm: A Case Report with Advanced Techniques
Source: Int J Mol Sci. 2021 May 30;22(11):5857. doi: 10.3390/ijms22115857 (PMC8199233; doi:10.3390/ijms22115857)
Supplement: Supplementary file 1 [file ijms-22-05857-s001.zip › Suppl Figure_01_GSEA_Cell motility.pptx]

## Slide 1
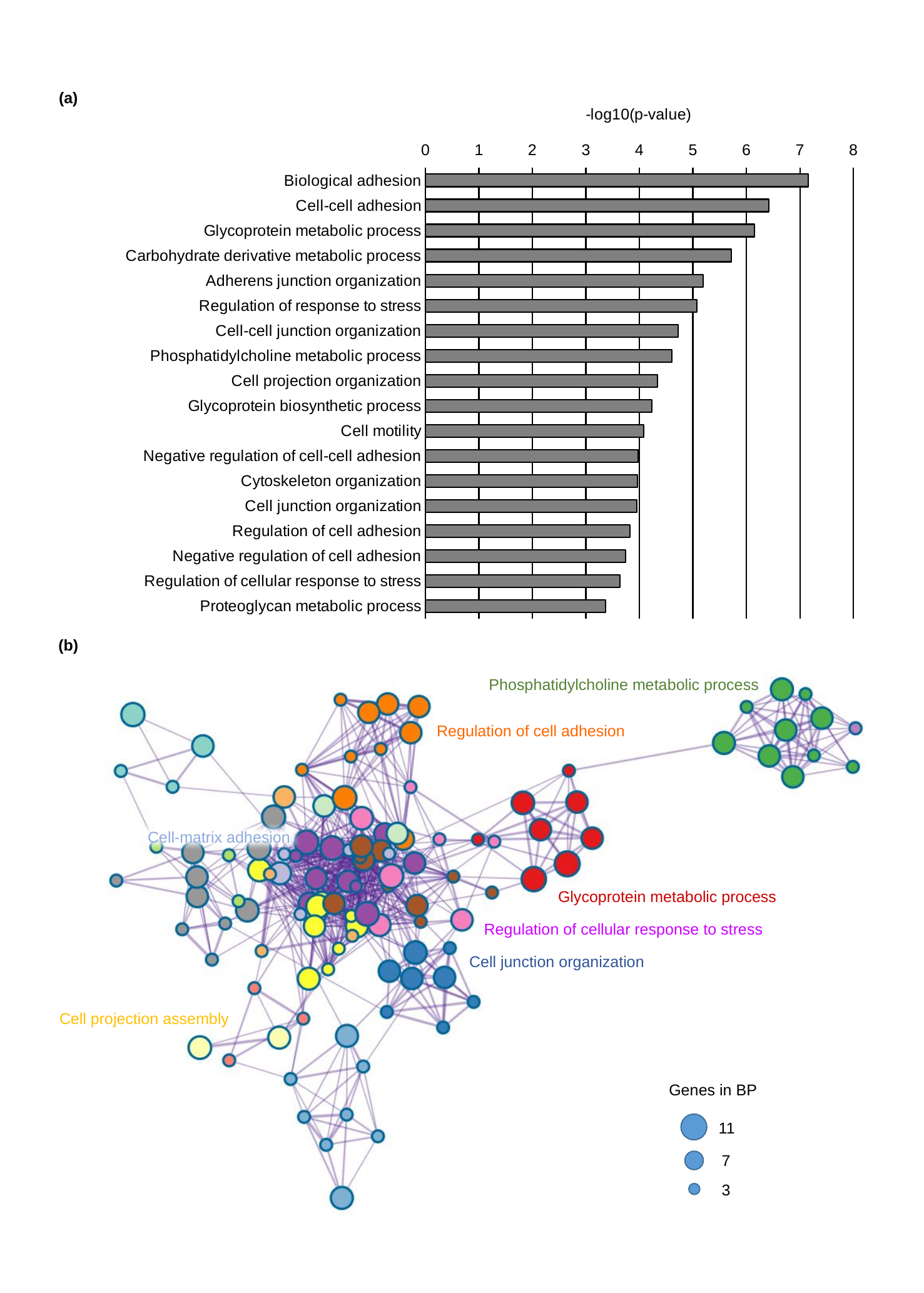

### Chart
| Category | |
|---|---|
| Biological adhesion | 7.1598939055432425 |
| Cell-cell adhesion | 6.422508200162775 |
| Glycoprotein metabolic process | 6.154901959985743 |
| Carbohydrate derivative metabolic process | 5.714442690992226 |
| Adherens junction organization | 5.189767482004916 |
| Regulation of response to stress | 5.071092309756048 |
| Cell-cell junction organization | 4.721246399047171 |
| Phosphatidylcholine metabolic process | 4.610833915635467 |
| Cell projection organization | 4.337242168318426 |
| Glycoprotein biosynthetic process | 4.229884705212898 |
| Cell motility | 4.081445469449727 |
| Negative regulation of cell-cell adhesion | 3.9788107009300617 |
| Cytoskeleton organization | 3.962573502059376 |
| Cell junction organization | 3.9507819773298185 |
| Regulation of cell adhesion | 3.8297382846050425 |
| Negative regulation of cell adhesion | 3.7423214251308154 |
| Regulation of cellular response to stress | 3.642065152999546 |
| Proteoglycan metabolic process | 3.3716110699496884 |(a)
(b)
Phosphatidylcholine metabolic process
Regulation of cell adhesion
Cell-matrix adhesion
Glycoprotein metabolic process
Regulation of cellular response to stress
Cell junction organization
Cell projection assembly
Genes in BP
11
7
3
